# Value of Contrast-Enhanced Ultrasound and Acoustic Radiation Force Impulse Imaging for the Differential Diagnosis of Benign and Malignant Thyroid Nodules

**DOI:** 10.3389/fphar.2018.01363

**Published:** 2018-11-27

**Authors:** Yan He, Xiao Yan Wang, Qiao Hu, Xue Xue Chen, Bing Ling, Hai Ming Wei

**Affiliations:** ^1^Department of Ultrasound, The People’s Hospital of Guangxi Zhuang Autonomous Region, Nanning, China; ^2^Department of Pathology, The People’s Hospital of Guangxi Zhuang Autonomous Region, Nanning, China

**Keywords:** thyroid nodule, contrast-enhanced ultrasound, acoustic radiation force impulse-imaging, shear wave velocity, receiver operating characteristic curve

## Abstract

**Objectives:** To assess the value of contrast-enhanced ultrasound (CEUS) and acoustic radiation force impulse (ARFI) imaging for the differential diagnosis of benign and malignant thyroid nodules.

**Methods:** CEUS was performed in eighty-eight thyroid nodules. The patterns of CEUS were analyzed, and ARFI was then performed. The shear wave velocities (SWVs) of the nodules and the surrounding normal thyroid tissue were obtained. The areas under the curve (AUCs) and cut-off value were obtained by a receiver operating characteristic (ROC) curve analysis. The sensitivity, specificity, positive predictive value (PPV), negative predictive value (NPV) and diagnostic rate of each modality were assessed and compared using pathological diagnosis.

**Results:** Among 88 nodules, 29 nodules were malignant and 59 were benign. The sensitivity, specificity, PPV, NPV, and diagnostic rate of CEUS were 79.3, 91.5, 82.1, 90, and 87.5%, respectively. Using a cut-off value of 2.565 m/s for SWV, the sensitivity, specificity, PPV, NPV and diagnostic rate for malignancy were 75.9, 94.9, 88.0, 88.9, and 88.6%, respectively. The AUC was 0.878. The sensitivity, specificity, PPV, NPV and diagnostic rate of CEUS in combination with ARFI were 93.1, 89.8, 81.8, 96.3, and 90.9%, respectively.

**Conclusion:** Both CEUS and ARFI are valuable for the differential diagnosis of benign and malignant thyroid nodules. Combining these two methods can improve the diagnostic rate.

## Introduction

Thyroid nodules are not specific to a particular disease but are a common clinical manifestation of various thyroid diseases. Only 4∼7% of adult cases can be found by palpation of the thyroid nodules (Vander et al., 1968), but up to 50∼ 67% can be found by conventional ultrasound examination (Mehanna et al., 2009). Most thyroid nodules are benign, and approximately 5% are malignant (Gharib, 1997). The incidence of thyroid cancer has increased in recent years (Paes et al., 2010). The degree of malignancy is low and the prognosis is good for most differentiated thyroid cancers, however, some thyroid cancers are invasive (Gilliland et al., 1997). Therefore, early detection, early diagnosis and early treatment are key in the prevention and treatment of thyroid malignant nodules.

Conventional ultrasound has become the preferred imaging examination method, but two-dimensional ultrasound overlaps to some degree between benign and malignant nodules. Therefore, the diagnosis of thyroid nodules by conventional ultrasound alone has some limitations (Hoang et al., 2007; Azar et al., 2013).

The development of contrast-enhanced ultrasound (CEUS) can be traced back to 1969. Gramiak et al. (1969) found using an aortic root injection of indocyanine blue that M echocardiography can present an echo enhancement phenomenon. Researchers later discovered that the phenomenon was due to the injection of liquid-containing microbubbles and began to search for a more ideal ultrasound contrast agent, leading to new prospects for the use of CEUS. The value of CEUS for evaluating liver lesions has been widely recognized (Chen et al., 2005; Seitz et al., 2010), and its applications in assessments of breast, thyroid, prostate and other organs have become a focus of research (Mitterberger et al., 2007; Friedrich-Rust et al., 2010). CEUS can dynamically respond to tissue perfusion in real time and has important clinical value for the diagnosis of thyroid nodules.

The concept of elastic imaging was proposed by Ophir et al. (1991). Traditional compression elastic imaging is applied by the operator according to the degree of tissue deformation after pressure to obtain the relative stiffness of the organization. However, traditional compression elastic imaging can easily be affected by the operating frequency, the intensity of the pressure, the size of the area of interest and the size of the lesion, among other factors. The technique has some shortcomings, including poor reproducibility and subjectivity (Luo et al., 2008). Acoustic radiation force impulse imaging (ARFI) includes virtual touch tissue imaging (VTI), and virtual touch tissue quantification (VTQ) (Nightingale et al., 2001, 2002, 2003). The parenchyma is characterized by a greater degree of microstructure, greater stiffness, less deformation and faster shear wave speed. The stiffness of thyroid nodules is related to their pathological structure. ARFI can directly reflect the stiffness of thyroid nodules and can provide information that can be used in the differential diagnosis of benign and malignant thyroid nodules. Many studies have reported differential diagnoses of benign and malignant thyroid nodules by CEUS or ARFI (Nemec et al., 2012; Calvete et al., 2014), but few reports have described the difference between these two tests in the diagnosis of thyroid nodules. This study aimed to study the characteristics of micro-blood flow perfusion and shear wave velocity (SWV) in benign and malignant thyroid nodules by CEUS and ARFI.

## Materials and Methods

### Patients

The study was approved by the Ethical Committee of the People’s Hospital of Guangxi Zhuang Autonomous Region, and written informed consent was obtained from all patients. Eighty-three patients were recruited from November 2013 to March 2017 (a total of 88 thyroid nodules); the patients were aged from 15 to 87 years with a mean age of (46.0 ± 15.2) years. The size of the nodules ranged from 7 to 66 mm, with an average of (28.7 ± 13.9) mm. All cases were confirmed by pathology.

Selection criteria: (1) Solid or cystic nodules with a solid fraction > 50%. Shear waves cannot spread the liquid. (2) A nodule diameter > 6 mm was required because the sampling frame size of ARFI is 5^∗^6 mm. The nodule size was set to be smaller than the sampling frame such that the sampling frame would include normal tissue surrounding the nodules. The results obtained for the sampling frame did not reflect the results for the nodule. (3) Normal thyroid tissue was present around the nodule for comparison. (4) No surgery, drug or chemotherapy was performed/administered before the operation. The pathological results were confirmed postoperatively.

### Instruments and Inspection Methods

CEUS was performed by using a GE E9 color Doppler ultrasound instrument equipped with a 15.0 MHz linear array transducer and a 9.0 MHz contrast transducer with a mechanical index of 0.08.

ARFI was performed by a Siemens ACUSON S2000 system equipped with a 9L4 line array probe, built-in ARFI imaging technology and the related software. The region of interest (ROI) was defined 3–40 mm from the probe surface, and the ROI was a fixed size of 5 × 6 mm.

The patient was placed in a supine position and told to breathe calmly; full exposure of the thyroid was obtained. The thyroid and bilateral neck were scanned using routine ultrasound. The position, number, size, shape, aspect ratio, boundary, internal echo, calcification type, and sound halo of the lesion and the presence or absence of neck lymph node enlargement were observed.

The patient was asked to breath calmly and avoid swallowing during the CEUS examination. The contrast agent used was Bracco SonoVue (Italy). Saline (5 mL) was added to the dry powder and mixed in an oscillating shaker. Six sulfur hexafluoride microbubble suspensions were configured. An appropriate section showing both the complete lesion and the surrounding normal tissue was selected. The channel was established in patients using the superficial elbow vein. Five milliliters of physiological saline was injected after a rapid bolus injection of 1.8 mL of the mixed contrast agent. The timer was then started, and the observation section remained unchanged. The image was dynamically observed after administration for 3 min. The imaging data were then stored. Contrast performance was observed, including the time required for contrast wash-in (earlier wash-in, wash-in at the same time, later wash-in), the pattern of contrast wash-in (concentric or non-concentric), ring enhancement or lack thereof, contrast intensity at the peak time (low enhancement, equal enhancement, or high enhancement), contrast-enhanced distribution (homogeneous enhancement or inhomogeneous enhancement) and contrast wash-out (earlier or not earlier).

The probe was vertically touched to the surface of the skin at the location of the target thyroid lesion, avoiding the pulsation of the carotid artery. The patient was asked to continue breathing calmly and avoid swallowing. VTQ imaging mode was then begun. The “Update” key was pressed to capture the image after it became stable. The SWV value was then recorded. Gross calcification, necrosis and cystic regions were avoided. Five VTQ results were obtained for the internal and peripheral normal tissues of each nodule. If the ROI was confirmed by CEUS to be a solid lesion (“X.XX m/s”), the ROI was considered too hard and beyond the scope of measurement (Tozaki et al., 2011b). This value was replaced by “9.00 m/s.” The average SWV value was recorded.

### Statistics Analysis

Statistical analyses were performed using SPSS software version 16.0. Quantitative data are expressed as the means ± SD or as a minimum–maximum range. The distribution of variables was analyzed using the K-S test. Groups were compared using the *t*-test. The effect of SWV on the diagnosis of thyroid cancer was evaluated based on the ROC curve, which was used to select the best critical value. The accuracy of the diagnostic test was evaluated based on the AUC and the diagnostic test evaluation method. Categorical data were compared using Fisher’s exact test or the χ^2^-test. *P*-values < 0.05 were considered to indicate significant differences.

## Results

### Pathological Results

In total, 88 thyroid nodules were confirmed by pathology, including 29 malignant and 59 benign nodules. Malignant nodules included 27 thyroid papillary carcinomas, 1 thyroid follicular carcinoma, and 1 thyroid metastasis from a nasopharyngeal carcinoma. Benign nodules included 35 nodular goiters, 15 nodular goiters with nodular hyperplasia, and 9 thyroid adenomas.

### CEUS Results

Eighty-eight contrast-enhanced ultrasound modes of thyroid nodules are shown in Table 1. Malignant nodules showed concentric and inhomogeneous low or equal enhancement, and earlier contrast wash-out. Benign nodules showed diffuse and ring enhancement. Inhomogeneous, low or equal enhancement has been used as an index for the diagnosis of malignant nodules based on contrast-enhanced ultrasound (Zhang B. et al., 2010).

**Table 1:** Contrast-enhanced ultrasound modes of thyroid nodules.

Pathology	Entry pattern			Regression pattern			Contrast pattern	
	Wash-in earlier	Wash-in at the same time	Wash-in later	Wash-out earlier	Wash-out at the same time	Wash-out later	Concentric	Non-concentric
Benign	23	29	7	9	40	10	6	53
Nodular goiter	12	16	7	7	25	3	6	29
Nodular goiter with nodular hyperplasia	4	11	–	2	11	2	–	15
Adenoma	7	2	–	–	4	5	–	9
Malignant	9	7	13	17	11	1	22	7
Papillary carcinoma	8	7	12	16	11	–	21	6
Follicular carcinoma	1	–	–	–	–	1	–	1
Metastasis carcinoma	–	–	1	1	–	–	1	–
χ^2^-value	12.607			17.101			38.677	
*P*-value	0.002			<0.001			<0.001	

### ARFI Results

The SWV values of the internal and peripheral tissues of 88 thyroid nodules and the SWV ratios of the internal and peripheral normal tissues of the lesions are presented in Table 2.

**Table 2:** ARFI results for thyroid nodules.

	Internal SWV value (m/s)	Surrounding SWV value (m/s)	SWV ratio
**Malignant**	5.81 ± 3.21 (range: 0.97∼9.00)	2.11 ± 0.45 (range: 1.24∼3.44)	2.74 ± 1.40 (range: 0.56∼5.57)
Papillary carcinoma	5.86 ± 3.15 (range: 1.24∼9.00)	2.10 ± 0.42 (range: 1.31∼3.44)	2.79 ± 1.41 (range: 0.56∼5.57)
Follicular carcinoma	1.65 ± 0.41 (range: 0.97∼2.00)	1.48 ± 0.21 (range: 1.24∼1.70)	1.12
Metastasis carcinoma	9.00	2.91 ± 0.23 (range: 2.55∼3.00)	3.09
**Benign**	2.00 ± 1.17 (range: 0.51∼9.00)	2.02 ± 0.40 (range: 1.16∼3.82)	1.02 ± 0.62 (range: 0.20∼4.78)
Nodular goiter	2.02 ± 1.38 (range: 0.51∼9.00)	2.01 ± 0.41 (range: 1.16∼3.32)	1.03 ± 0.73 (range: 0.20∼4.78)
Nodular goiter with nodular hyperplasia	2.13 ± 0.92 (range: 1.56∼8.40)	1.99 ± 0.31 (range: 1.50∼3.82)	1.08 ± 0.44 (range: 0.46∼2.41)
Adenoma	1.74 ± 0.51 (range: 1.05∼3.80)	2.12 ± 0.47 (range: 1.31∼3.23)	0.87 ± 0.35 (range: 0.40∼1.48)

There were significant differences in SWV values between the malignant nodules and the surrounding tissue (*P* < 0.05). There were also significant differences in SWV values between benign and malignant nodules (*P* < 0.05). There was no significant difference in SWV value between benign nodules and the surrounding tissue (*P* > 0.05), although there were significant differences in the SWV ratios between benign and malignant nodules (*P* < 0.05).

**FIGURE 1:**
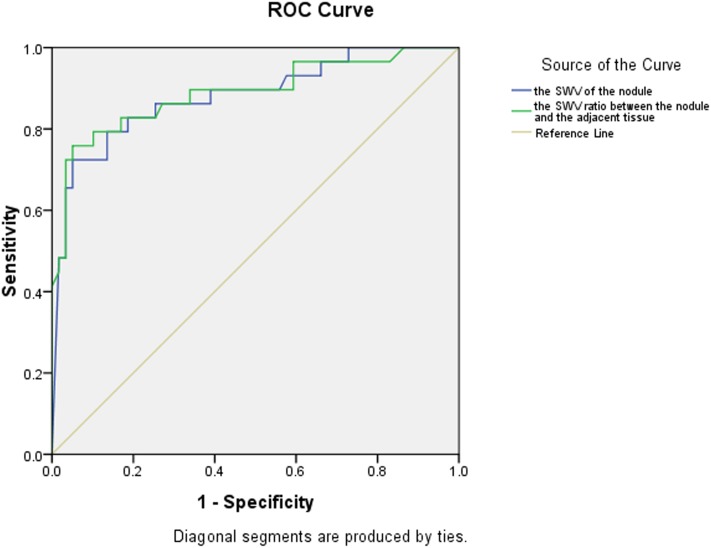
ROC curves of the SWV value and SWV ratio of thyroid nodules.

Using cut-off values of 2.565 m/s for SWV and 1.565 for the SWV ratio, the areas under the ROC curves were 0.878 and 0.875, respectively, for the diagnosis of malignant thyroid nodules (Table 3 and Figure 1).

**Table 3:** Area under the ROC curve for the SWV value and the SWV ratio of thyroid nodules.

	Under curve area	*P*-value	95% confidential interval	
			Lower limit	Upper limit
SWV value	0.878	<0.001	0.791	0.964
SWV ratio	0.875	<0.001	0.785	0.964

### Value of CEUS, ARFI and Their Combination for the Diagnosis of Benign and Malignant Thyroid Nodules

The diagnostic values of CEUS, ARFI and their combination in the diagnosis of thyroid nodules are shown in Table 4. There was no significant difference between CEUS and ARFI in terms of their diagnostic value regarding thyroid nodules (*P* > 0.05). However, there were significant differences in the diagnostic values of CEUS, ARFI and the combination of CEUS and ARFI (*P* < 0.05).

**Table 4:** Comparison of various CEUS and ARFI indices for the diagnosis of thyroid nodules.

		Pathology		Sensitivity	Specificity	Positive predictive value	Negative predictive value	Accuracy rate	Youden index
		Malignant Benign							
CEUS	Malignant	23	5	79.3%	91.5%	82.1%	90%	87.5%	0.708
	Benign	6	54						
ARFI (SWV)	Malignant	22	3	75.9%	94.9%	88.0%	88.9%	88.6%	0.708
	Benign	7	56						
Both	Both negative or one positive	27	6	93.1%	89.8%	81.8%	96.3%	90.9%	0.829
	Both negative	2	53						

## Discussion

Differences in benign and malignant thyroid nodules can be found in the boundary, internal echo, aspect ratio, calcification type, blood supply distribution characteristics and blood flow resistance index in conventional ultrasound. However, two-dimensional ultrasound images of benign and malignant nodules overlap to some extent. It is difficult to differentiate benign and malignant thyroid nodules using only conventional ultrasound, whereas CEUS can show microvascular perfusion in the nodules. Benign and malignant nodules can show different patterns of contrast. During ARFI, no external force is applied, potentially avoiding the influence of subjective factors. ARFI can be used to quantitatively evaluate tissue stiffness, yielding a specific numerical value. Tissue stiffness is often closely related to the underlying pathological structure, and malignant nodules often have greater tissue stiffness than benign nodules (Mandel, 2004).

### CEUS Modes Relating to Thyroid Nodules and Their Pathology

**FIGURE 2:**
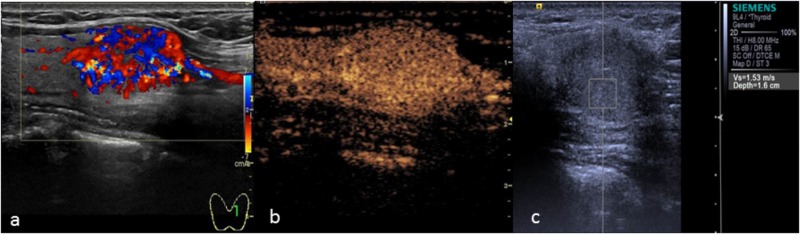
Papillary thyroid carcinoma. **(a)** Two-dimensional ultrasound showing a hypo-echoic nodule with calcification. **(b)** CEUS showing heterogeneous low enhancement. **(c)** The SWV value was X.XX m/s.

**FIGURE 3:**
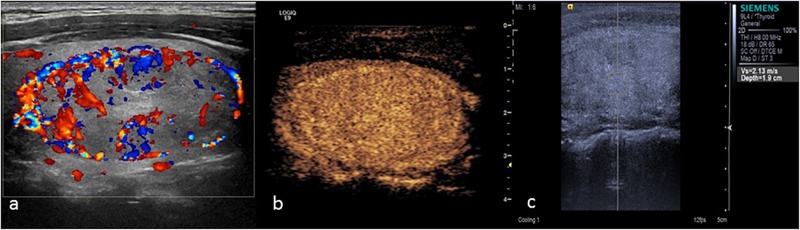
Papillary thyroid carcinoma. **(a)** Color Doppler ultrasound showing rich blood flow in the nodule. **(b)** CEUS showing heterogeneous homogeneous equal enhancement. **(c)** The SWV value was 1.53 m/s.

Zhang B. et al. (2010) prospectively observed CEUS modes for thyroid nodules and proposed that inhomogeneous enhancement is an important indicator that can be used in the diagnosis of malignant nodules. In the malignant group of this study, most thyroid papillary carcinomas showed concentric and inhomogeneous enhancement (Figure 2). The reasons for this may be as follows: (1) Malignant nodules secrete vascular endothelial growth factor (VEGF), which stimulates the growth of new blood vessels within the nodule and adjacent tissues. Neovascularization comprises tortuous and complicated vessels with uneven diameters, eccentric distributions and irregular vascular branches (Zhang Y. et al., 2010). (2) Microcalcification exists in thyroid papillary carcinoma, which influences neovascularization. This further aggravates the uneven distribution of blood vessels. The vascular space distribution of malignant nodules is complex. Angiogenesis can be divided into forms that are present in marginal and central areas. Blood vessels are relatively dense in marginal areas but are sparse in central areas. Differences in vascular density between marginal and central areas may cause concentric enhancement, in accordance with the results reported by Hornung et al. (2012) that the AUC of the TIC of the contrast agent in the marginal zone of the nodules was higher than that in the central area. Because of the arteriovenous fistula in malignant nodules, the contrast agent washed out earlier. Thyroid papillary carcinoma showed low enhancement, equal enhancement, and high enhancement in the malignant group, which may be related to the size and vascular density of the nodules. Molinari et al. (2010) proposed that the vascular density of thyroid malignant nodules is higher than that of benign nodules, while Moon et al. (2010) found that internal flow is more likely to occur in benign nodules, and the lack of blood supply in the nodules may be related to malignancy. These findings reflect the complexity of the blood supply of malignant thyroid nodules. Thyroid papillary carcinoma may exhibit a lack of blood supply or a rich blood supply (Figure 3).

**FIGURE 4:**
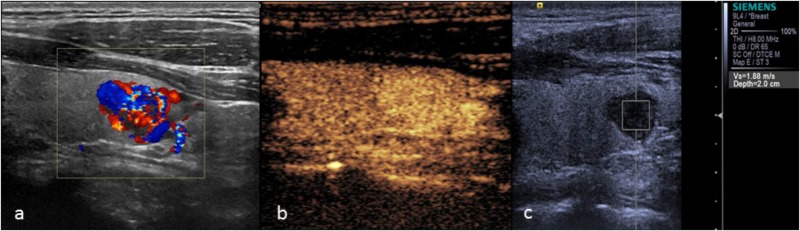
Thyroid follicular carcinoma. **(a)** Color Doppler ultrasound showing rich blood flow in the nodule. **(b)** CEUS showing heterogeneous homogeneous high enhancement. **(c)** The SWV value was 1.88 m/s.

There was 1 case of follicular carcinoma in the malignant group (Figure 4). CEUS showed a fast wash-in, a slow wash-out, and homogeneous and high enhancement, which was similar to the CEUS results for adenoma, without ring enhancement. However, CEUS cannot distinguish between thyroid follicular carcinoma and follicular adenoma based on the mode of growth, envelope thickness or cytological features. The only pathological diagnostic criterion of follicular carcinoma was tumor invasion of the envelope or blood vessels. Due to infiltration of the surrounding tissue by the tumor in follicular carcinoma, CEUS showed no ring enhancement, which is caused by the expansive growth of the adenoma, leading to extrusion of the peripheral blood vessels.

**FIGURE 5:**
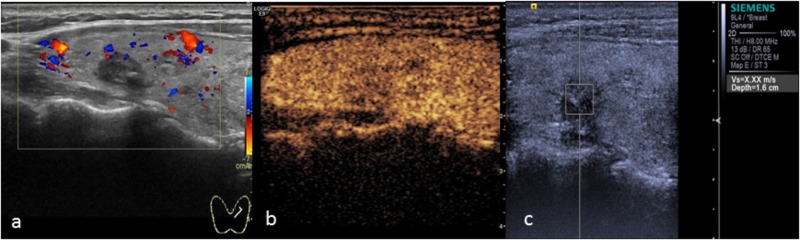
Thyroid adenoma. **(a)** Color Doppler ultrasound showing circumferential blood flow around the nodule. **(b)** CEUS showing ring enhancement. **(c)** The SWV value was 2.13 m/s.

**FIGURE 6:**
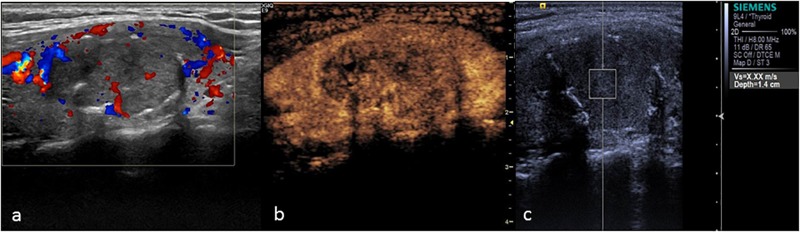
Nodular goiter. **(a)** Color Doppler ultrasound showing a hypo-echoic nodule with coarse calcification. **(b)** CEUS showing heterogeneous low enhancement. **(c)** The SWV value was X.XX m/s.

The main manifestation of benign nodules was ring enhancement (Figure 5). Thyroid adenoma is derived from follicular epithelial cells. Benign tumors show expansive growth with complete capsules. Blood vessels are gradually extruded to the surroundings of the tumor during the growth process, forming rich encircled blood vessels. During the process of repeated hyperplasia and nodular goiter repair, the nodule extrudes to the surrounding thyroid tissue and forms a peripheral vascular ring. Therefore, CEUS examination of most benign nodules showed ring enhancement. Various contrast patterns of nodular goiter were observed, and most showed wash-in and wash-out at the same time as the surrounding thyroid tissue. Zhang H.L. et al. (2013) showed that nodular goiters underwent different stages of hyperplasia and repair. The distribution of blood vessels within the nodules was different at different stages of the disease, ultimately yielding a range of CEUS performance levels. In this study, 5 cases of nodular goiter were misdiagnosed as thyroid carcinoma because of inhomogeneous low enhancement (Figure 6).

### ARFI Results for Thyroid Nodules and Their Pathology

The VTQ technique quantitatively measures tissue stiffness by calculating the SWV during tissue deformation; the greater the SWV value is, the harder the tissue. Benign thyroid lesions, such as thyroid adenoma or nodular goiter, are mainly composed of follicular cells of different sizes containing a large amount of colloidal components. The texture of the tissue is soft, whereas malignant thyroid lesions are harder. For example, nipple branches and fibrovascular stroma are found in thyroid papillary cancer, with psammoma bodies concentrically arranged in the stroma and greater hardness.

The results of this study showed that the SWV value of benign nodules was close to that of the surrounding normal tissue. Malignant thyroid nodules were harder than benign nodules, which were in turn harder than the surrounding normal tissues. The average SWV value was 5.81 m/s. To account for individual differences, we measured the ratio between the SWV value of the lesion and the surrounding normal tissue. The SWV ratio of malignant nodules was 2.74 ± 1.40, which was significantly higher (*P* < 0.05) than that of benign nodules (1.02 ± 0.62). This finding suggests that the texture of malignant nodules was harder than that of benign nodules. Using a cut-off SWV value of 2.565 m/s for the diagnosis of thyroid malignant nodules, the area under the ROC curve was 0.878, similar to the result reported by Hamidi et al. (2015). We concluded that 2.66 m/s was the optimum cut-off point. In the malignant group, the SWV values of 13 thyroid cancers were found to be “X.XX m/s” in repeated tests (Figure 2). CEUS confirmed the nodule to be a solid lesion, whose stiffness exceeded the scope of detection using this instrument. The output of “X.XX m/s” when measuring SWV for a solid nodule was highly suggestive of malignancy. This finding is similar to the results of Tozaki et al. (2011a) and Fukuhara et al. (2014). However, not all nodules yielding “X.XX m/s” were malignant. “X.XX M/s” appeared in the SWV measurement of 1 nodular goiter (Figure 6), possibly indicating that the nodule was found during the late stage of the disease. The occurrence of fibrosis, degenerative changes or a large number of calcium salt deposits hardened the nodule, causing the appearance of “X.XX M/s.” The mean SWV value of 1 thyroid follicular carcinoma was 1.65 m/s. The SWV ratio of the nodule to the peripheral normal tissue was 1.12, suggesting that the texture of the nodule was soft. This finding may be related to the pathological characteristics of thyroid follicular carcinoma. Except in cases of infiltration of the envelope or the blood vessels, thyroid follicular carcinoma is composed of follicles of different sizes, and the texture is no different from that of thyroid follicular adenoma. Therefore, the SWV value for thyroid follicular carcinoma may not be higher than that for benign nodules or the surrounding normal tissue.

### Value of CEUS and ARFI for the Diagnosis of Thyroid Nodules

In this study, the sensitivities of CEUS and ARFI for the diagnosis of thyroid malignant nodules were 79.3 and 75.9%, respectively. To reduce the rate of misdiagnosis, CEUS and ARFI can be combined for the diagnosis of thyroid malignant nodules, increasing the sensitivity to 93.1%. This difference was statistically significant (*P* < 0.05). Although the specificity of the combination of CEUS and ARFI for the diagnosis of thyroid malignant nodules was lower, the Youden index was increased to 0.829. The Youden index combines information regarding sensitivity and specificity and is used to evaluate the authenticity of a test. Larger values of the index indicate a better effectiveness of the test and a higher authenticity. Combining the CEUS and ARFI tests yielded a higher diagnostic value for the diagnosis of thyroid nodules than either of the tests alone.

This study has several limitations. First, the sample size of this study was small. Nodules with a diameter of less than 6 mm were excluded from this study. Thus, the study cannot provide information about thyroid microcarcinoma. Second, only one pathological type was observed in the malignant group. Most of the nodules were papillary thyroid cancers; only 1 follicular carcinoma and 1 metastatic cancer were included. Finally, “X.XX m/s” was output for several cases for the measurement of papillary thyroid cancer, and this value was replaced by “9 m/s.” The actual SWV value of these papillary thyroid cancer was likely much larger than “9 m/s.” Thus, the SWV value of papillary thyroid cancercould have been underestimated. It is necessary to expand on this research in the future by increasing the sample size, increasing the number of pathological types and using real-time shear wave elastic imaging.

## Conclusion

To conclude, CEUS and ARFI were of value for the differential diagnosis of benign and malignant thyroid nodules. The combination of these techniques can improve the accuracy of the diagnosis.

## Author Contributions

XW and QH conceived and designed the experiments. YH and QH performed the experiments. YH analyzed the data. BL, XC, and HW contributed reagents, materials, and analysis tools. QH and YH wrote the paper.

## Conflict of Interest Statement

The authors declare that the research was conducted in the absence of any commercial or financial relationships that could be construed as a potential conflict of interest.
